# Comparison of opioid-free versus weak-opioid general anesthesia on quality of postoperative recovery in soldiers undergoing arthroscopic meniscal surgery

**DOI:** 10.3389/fmed.2025.1665123

**Published:** 2025-10-20

**Authors:** Xikun Sun, Xin Ding, Xiaohang Han, Yue Wang, Xuli Cheng, Lin Li

**Affiliations:** 1 Department of Anesthesiology, General Hospital of Northern Theater Command, Shenyang, Liaoning, China; 2 Department of Anesthesiology, General Hospital of Northern Theater Command, Shenyang, Liaoning, China; 3 Graduate School, Dalian Medical University, Dalian, Liaoning, China

**Keywords:** opioid-free anesthesia, weak opioid anesthesia, QoR15 scores, soldier, meniscus surgery

## Abstract

**Objective:**

To determine whether opioid-free anesthesia improves early postoperative recovery compared with weak-opioid anesthesia in soldiers undergoing meniscal surgery for training-related injuries.

**Method:**

A total of 100 patients scheduled for elective meniscal surgery were randomized into two groups (*n* = 50 each): weak-opioid anesthesia group (WOA) and opioid-free anesthesia (OFA) group. Anesthesia induction consisted of alfentanil 20 μg/kg in the WOA group and esketamine 0.2 mg/kg in the OFA group. Intraoperatively, the OFA group received esketamine 0.2 mg/kg/h, lidocaine 1 mg/kg/h, and sevoflurane (MAC 1.0–1.4). The WOA group received remifentanil 0.1 μg/kg/h and sevoflurane (MAC 0.8–1.0). The primary endpoint was the QoR15 score at 24 h postoperatively.

**Results:**

The OFA group achieved significantly higher QoR15 scores at 24, 48, and 72 h, with the 24-h difference exceeding the threshold for clinical significance. The OFA group was associated with longer awakening times but earlier return of gastrointestinal function (shorter time of flatus). Intraoperatively, the WOA group experienced greater reductions in heart rate and mean arterial pressure, with a higher incidence of remarkable bradycardia. Postoperatively, the OFA group reported lower NPRS scores across the first 3 days, required less rescue analgesia, and had a lower incidence of rebound pain.

**Conclusion:**

Compared with weak-opioid anesthesia, opioid-free anesthesia significantly improves early postoperative recovery quality, as measured by the QoR15, in soldiers undergoing arthroscopic meniscal surgery.

## Background

Lower-extremity injuries affect approximately one-quarter of American soldiers, with meniscal injuries being the most common subtype (1, 2). General anesthesia is often preferred for knee arthroscopy because it mitigates perioperative anxiety and avoids discomfort related to surgical positioning (3). Opioids, although traditionally integral to general anesthesia, are associated with various side effects such as opioid-induced hyperalgesia, nausea, and vomiting, which may prolong recovery and contribute to greater socioeconomic burden (4). To minimize these drawbacks, most anesthesiologists adopt multimodal analgesia and balanced anesthesia strategies, which provide adequate anesthetic depth and optimal surgical conditions while limiting drug-specific adverse effects (5). Opioid-free anesthesia (OFA) (6, 7), an extension of multimodal analgesia and balanced anesthesia (8) eliminates intraoperative opioid use by combining non-opioid intravenous medications with nerve-blocking techniques. Multiple studies have demonstrated that opioid-free anesthesia accelerates postoperative recovery in breast surgery (9), thyroidectomy (10), and laparoscopic cholecystectomy (11), findings consistent with the Enhanced Recovery After Surgery (ERAS) principles. The Quality of Recovery-15 (QoR15) score, which is a clinically meaningful study endpoint and has similar evaluation validity to the QoR40 (12), can assess postoperative recovery quality in five dimensions (13): physical comfort, physical independence, emotional state, psychological support, and pain. As soldiers have unique vocations, it is imperative that patients recuperate and rejoin the military as soon as possible following surgery. To date, no studies have explored the effects of OFA in soldiers undergoing meniscal surgery. This study aimed to investigate whether OFA could enhance the quality of rehabilitation following arthroscopic meniscal surgery in troops.

## Methods

### Ethics statement

This study was approved by the Institutional Review Board of General Hospital of Northern Theater Command [Y(2024)146] and registered in the Chinese Clinical Trial Registry (ChiCTR2400092713). The trial adhered to the Declaration of Helsinki, and informed consent was obtained from all patients.

### Patients

This was a single-center, double-blind, randomized controlled study. A total of 100 patients were included in this study, which was conducted between December 2024 and March 2025 at General Hospital of Northern Theater Command. Patients were randomly assigned to either the opioid-free anesthesia group or the weak-opioid anesthesia (WOA) group in a 1:1 ratio using a computer-generated random number sequence. All surgical procedures were performed by the same team of surgeons, anesthesiologists, and nurses, who were not blinded to group allocation. To maintain blinding, independent anesthesiologists uninvolved in clinical care generated the random sequence, collected perioperative data, and remained unaware of patient allocation. Patients were blinded to their group assignments.

Eligible participants were military training-related injury patients aged 18–60 years, with a BMI between 18 and 28 kg/m^2^, and classified as ASA I or II, undergoing elective arthroscopic meniscal surgery. Exclusion criteria were: (1) history of allergies to anesthesia drugs or contraindication to esketamine, (2) severe hypertension or arrhythmia, (3) long-term use of opioids or nonsteroidal drugs, and (4) psychiatric illnesses preventing cooperation.

### Study design

Patients fasted for 6–8 h and were dehydrated for 2 h. Routine noninvasive monitoring (electrocardiography, noninvasive blood pressure, and pulse oximetry) was applied to all patients upon admission to the operating room. Distal adductor canal blocks were performed on the affected leg in both groups. A low-frequency convex array probe (TUO Ren, Henan, China) was placed on the anteromedial aspect of the distal thigh, approximately 6 cm proximal to the patellar base, corresponding to the anatomically defined adductor hiatus. From this location, the probe was advanced along the femoral artery until the femoral artery and vein were visualized within the adductor canal, between the vastus medialis and the adductor magnus muscles. Using an in-plane approach, the needle was inserted laterally and advanced through the vastus medialis muscle. When the needle reached the proximity of the saphenous nerve, 2 mL of normal saline was administered for hydrodissection. After confirming correct needle tip placement, 15 mL of 0.25% ropivacaine hydrochloride solution (Qilu Pharmaceutical Co., Ltd.) was injected. In the OFA group, 0.6 μg/kg dexmedetomidine (Sinopharm China National Pharmaceutical Co., Ltd.) was infused for 10 min before anesthesia induction. All patients underwent preoxygenation for 3 min at a flow rate of 6 L/min before anesthesia induction. Both groups received induction with 2 mg/kg propofol, 1 mg/kg lidocaine (Shandong Hualu Pharmaceutical Co., Ltd.), 5 mg dexamethasone (Zhejiang Xianju Pharmaceutical Co., Ltd.), and 0.2 mg/kg mivacurium chloride (Jiangsu Nhwa Pharmaceutical Co., Ltd.). In addition, the OFA group received 2 mg/kg esketamine (Jiangsu Hengrui Pharmaceutical Co., Ltd.), while the WOA group received 20 μg/kg alfentanil (Yicahng Humanwell Pharmaceutical Co., Ltd.). A laryngeal mask (Nanchang Biotek Medical Technology Co., Ltd.) was placed 3 min after injection of the neuromuscular blocking agent. Mechanical ventilation was initiated with a tidal volume of 7 mL/kg and a respiratory rate of 12 breaths/min. After laryngeal mask placement, the respiratory rate was adjusted to maintain end-tidal carbon dioxide at 35–45 mmHg. When the peak airway pressure was ≥25 mmHg, an additional one-third induction dose of mivacurium chloride was administered. Anesthesia maintenance in the OFA group consisted of esketamine 0.2 mg/kg/h, lidocaine 1 mg/kg/h, dexmedetomidine 0.2 μg/kg/h, and sevoflurane (Jiangsu Hengrui Pharmaceutical Co., Ltd.) at MAC 1–1.4, while the WOA group received remifentanil (Yichang Humanwell Pharmaceutical Co., Ltd.) 0.1 μg/kg/h, dexmedetomidine 0.2 μg/kg/h, and sevoflurane at MAC 0.8–1.0. Vasoactive drugs were administered when the mean arterial pressure (MAP) varied by more than 20% from baseline. Twenty minutes prior to surgical completion, the administration of esketamine, dexmedetomidine, and sevoflurane was discontinued, and propofol infusion (4 mg/kg/h) was continued until the end of the procedure. Flurbiprofen axetil (50 mg) and ondansetron (4 mg) were administered for postoperative analgesia and antiemesis, respectively. After the procedure, patients were transferred to the post-anesthesia care unit (PACU). As a remedial antiemetic, 4 mg of ondansetron was administered again if needed. When the Numeric Pain Rating Scale (NPRS) score was ≥3, 100 mg of tramadol was administered intramuscularly. Patients were transferred to the surgical ward once the steward post-anesthetic recovery score reached ≥ 4. The routine postoperative analgesic regimen consisted of loxoprofen sodium 100 mg/day. If a patient’s NPRS score in the surgical ward was ≥3, 100 mg of tramadol was administered intramuscularly as remedial analgesia.

### Outcomes

The primary outcome was the QoR15 score 24 h after surgery, a validated assessment tool comprising 15 items scored from 0 (poor recovery) to 10 (excellent recovery).

Secondary outcomes included QoR15 scores at 48 and 72 h postoperatively. The NPRS score, incidence of postoperative nausea and vomiting (PONV), and dizziness were recorded in the PACU at 24, 48, and 72 h postoperatively. Upon leaving the PACU, the 4’A’s Test (4AT) and Steward Post-Anesthetic Recovery Score were documented. Rebound pain, defined as an increase in NPRS score from ≤3 to ≥7 within 24 h after nerve block, was also assessed. Data on rescue analgesia, rescue antiemetics, and time to first flatus after surgery were collected. Heart rate (HR), mean blood pressure, and blood oxygen saturation were measured before anesthesia (T1), after laryngeal mask insertion (T2), during skin incision (T3), and at the end of surgery (T4). Remarkable bradycardia was defined as HR ≤ 40 beats/min, and hypotension was defined as a blood pressure below 80% of baseline or a systolic blood pressure below 90 mmHg. Delayed emergence was defined as failure to regain consciousness within 30 min of discontinuing medication and inability to make purposeful responses or actions to speech or stimuli.

### Statistical analysis

Each group included 20 patients in the pre-experimental phase. The average QoR15 score of the WOA group was 118.95 ± 4.39, and that of the OFA group was 125.15 ± 7.92 at 24 h after surgery. To minimize type I error and false positives, α was set at 0.025 (two-sided). Subsequently, PASS15 software was used, assuming α = 0.025, 1 − β = 0.9, and *σ* = 7.92. The calculated sample size was 41 patients per group. According to Myles et al., the minimal clinically important difference in the QoR15 score is 6 (14) which is less than the difference between the two groups in the pre-experiment. Therefore, allowing for a 20% dropout rate, the final study protocol planned to recruit 50 participants per group.

Statistical analysis was performed using SPSS software (version 25.0). Data are presented as mean ± standard deviation, median, numbers, or frequencies, as appropriate. Continuous variables with normal distribution and equal variance, such as BMI, were compared using independent sample t-tests. Normality of data distribution was assessed with the Shapiro–Wilk test, and homogeneity of variance was verified using the F-test. For non-normally distributed data, including demographic characteristics (e.g., age) and perioperative parameters (e.g., surgery duration, anesthesia duration, wake-up time, time to first flatus, 4AT score at PACU discharge, and NPRS), the Mann–Whitney U test was used. Categorical variables were analyzed using the Pearson chi-square test (e.g., ASA classification, remarkbale bradycardia, dizziness, and rescue analgesia) or Fisher’s exact test, where appropriate (e.g., hypotension, delayed recovery, PONV, rebound pain, and rescue antiemetic). A two-sided *p*-value < 0.05 was considered statistically significant.

## Results

**Figure 1:**
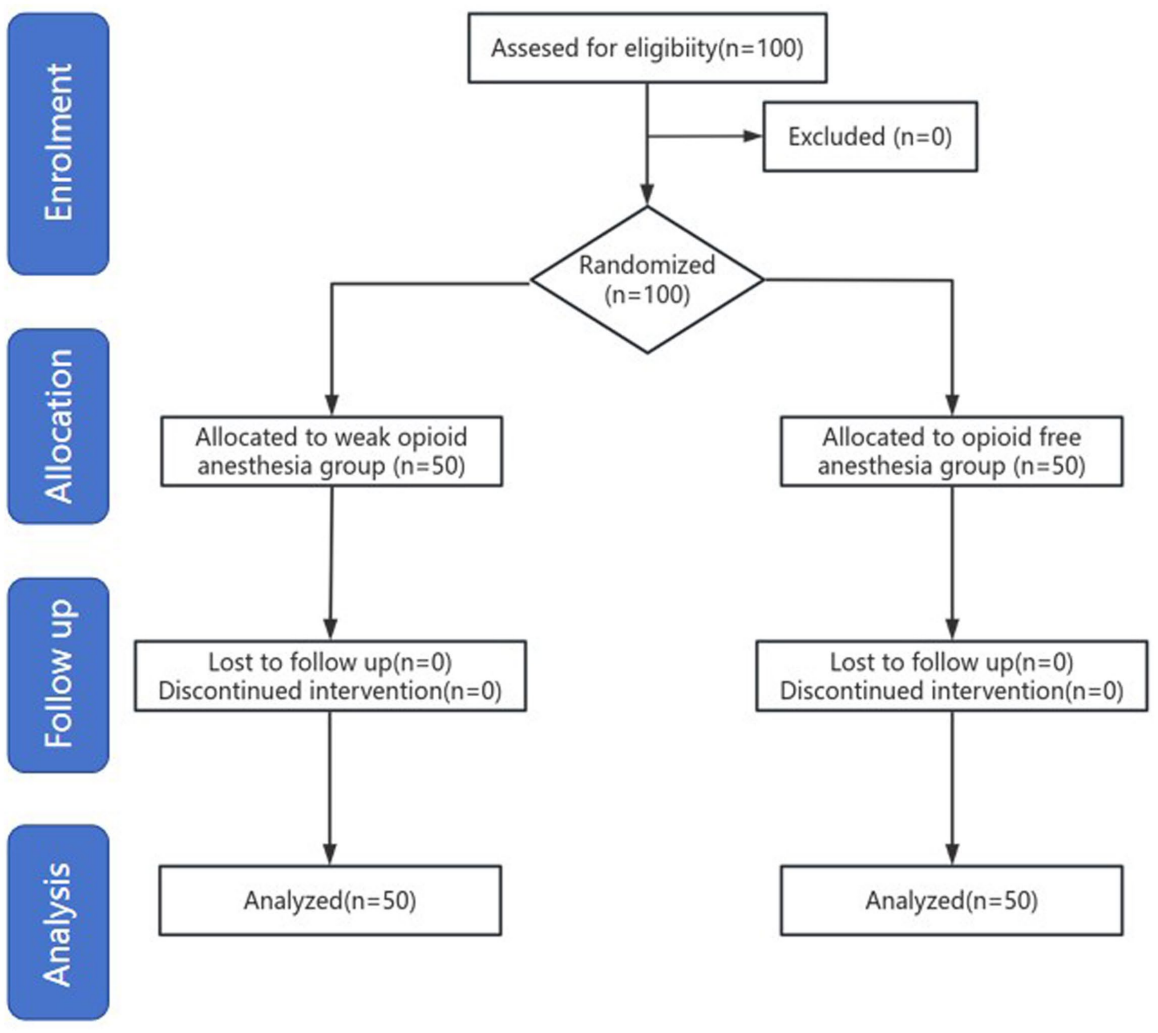
CONSORT diagram showing patient recruitment and follow up.

This study included 100 participants, all of whom completed the postoperative follow-up, with no withdrawals (Figure 1). The baseline characteristics of the two groups were similar. The duration of anesthesia, surgical duration, and 4AT scores were also comparable between groups. The wake-up time in the OFA group was significantly longer than that in the WOA group, whereas the time to first flatus was significantly shorter (Table 1).

**Table 1:** Demographics and procedure features.

	OFA group (*n* = 50)	WOA group (*n* = 50)	*P*-value	95% CI
Male, *n* (%)	50 (100%)	50 (100%)		
Age (years)	28 (23.75, 32.25)	29 (24, 33)	0.198	−4.546, 0.626
BMI (kg/m^2^)	24.11 ± 1.68	23.89 ± 1.81	0.855	−4.771, 0.909
ASA	23/37	25/25	0.689	1.005, 1.225
Surgery duration (min)	75 (65, 85)	75 (65, 87.5)	0.551	−10.564, 3.564
Anesthesia duration (min)	100 (85, 111)	100 (88.75, 115)	0.397	−12.590, 4.590
Wake up duration (min)	20 (17, 23)	14 (12, 15)	0.000***	5.311, 8.369
Postoperative first flatus (h)	11 (9, 12.25)	12 (10, 14)	0.03*	−2.416, −0.224
4AT at PACU discharge	0 (0, 0)	0 (0, 0)	0.31	−0.038, 0.118

BMI, Body Mass Index; PACU, Postanesthesia care unit. *P < 0.05; **P < 0.01; ***P < 0.001.

**Figure 2:**
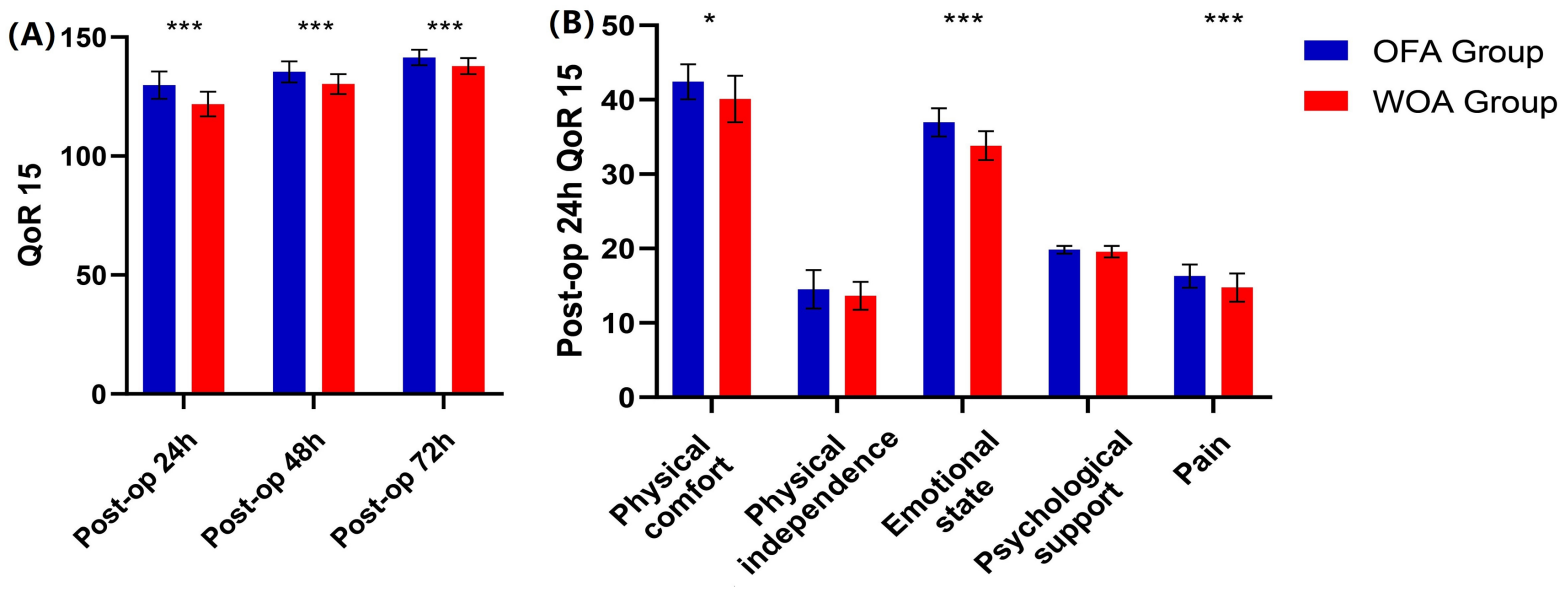
QoR15 score. Post-op: postoperative. *P < 0.05, **P < 0.01, ***P < 0.001.

The median QoR15 score at 24 h postoperative was significantly higher in the OFA group than in the WOA group (129 vs. 122). Subdomain analysis also showed higher scores in the OFA group for physical comfort (43 vs. 41), emotional state (37 vs. 34), and pain (17 vs. 15) at 24 h. QoR15 score remained significantly higher in the OFA group at 48 and 72 h postoperatively compared with the WOA group (135 vs. 130, 141 vs. 138, respectively; *p* < 0.05; Figure 2).

**Figure 3:**
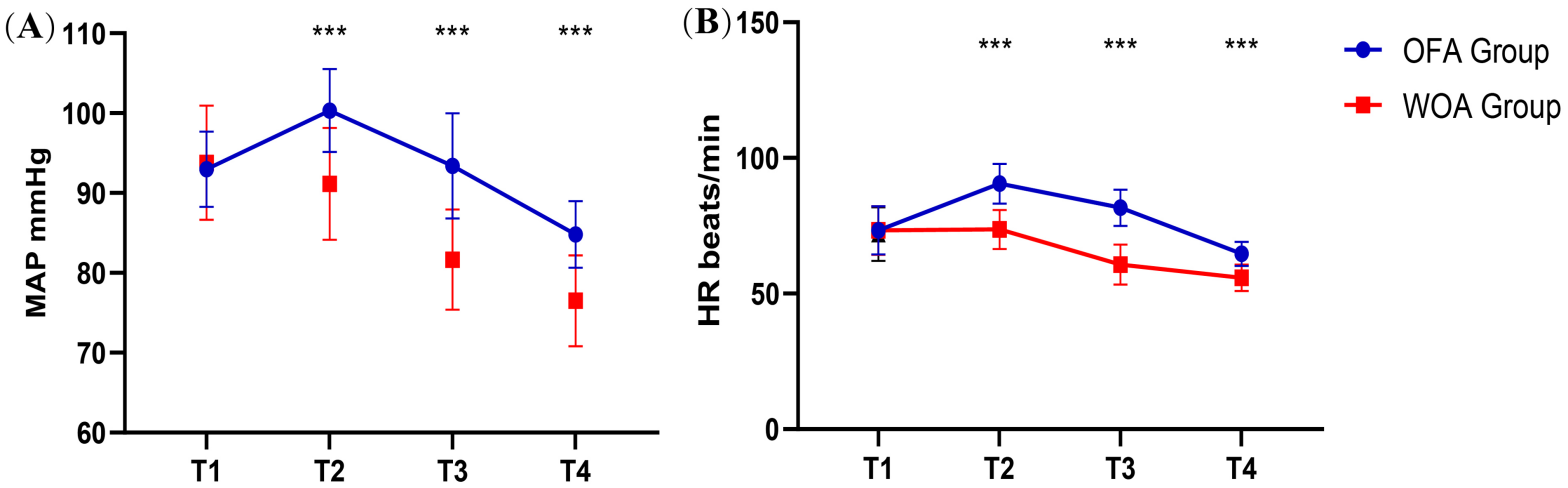
MAP and HR at T1, T2, T3 and T4. Pre-anesthesia (T1), after laryngeal mask insertion (T2), during skin incision (T3), and at the end of surgery (T4). *P < 0.05, **P < 0.01, ***P < 0.001.

No difference in HR and MAP between the groups at T1 was observed. However, HR and MAP in the OFA group were significantly higher than in the WOA group at T2, T3, and T4 (Figure 3).

The median NPRS score in the PACU was 1 in both the OFA and WOA groups, but scores differed significantly at 24 h (2.52 vs. 3.14), 48 h (1.70 vs. 2.44), and 72 h (1.06 vs. 1.62) postoperatively. In addition, the incidence of rebound pain and the rate of rescue analgesia use were lower in the OFA group (*p* > 0.05; Table 2).

**Table 2:** NPRS, rebound pain, rescue analgesia, and rescue antiemetic.

	OFA group (*n* = 50)	WOA group (*n* = 50)	*P*-value	95% CI
NPRS at PACU	1 (1, 1.25)	1 (1, 2)	0.587	−0.303, 0.183
NPRS at 24 h	2 (2, 3)	3 (2, 4)	0.003**	−1.001, −0.238
NPRS at 48 h	2 (1, 2)	2 (2, 3)	0.000***	−1.025, −0.454
NPRS at 72 h	1 (1, 1)	1 (1, 2)	0.000***	−0.795, −0.324
Rebound pain, *n* (%)	1 (2%)	2 (4%)	1.000	0.043, 5.582
Rescue analgesia, *n* (%)	4 (8%)	8 (16%)	0.218	0.128, 1.627
Rescue antiemetic, *n* (%)	2 (4%)	5 (10%)	0.436	0.069, 2.031

NPRS, Numeric Pain Rating Scale. *P < 0.05; **P < 0.01; ***P < 0.001.

The number of patients with remarkable bradycardia was higher in the WOA group (12% vs. 0%, 22% vs 6% *p* < 0.05). The incidence of intraoperative hypotension in the WOA group was six times higher than that in the OFA group (6% vs. 1%, *p* > 0.05). Three patients in the OFA group experienced delayed emergence (3 vs. 0, *p* > 0.05). The incidence of dizziness, nausea, and vomiting was comparable between groups (Table 3).

**Table 3:** Remarkable bradycardia, hypotension, delayed recovery, dizziness, and PONV.

	OFA group (*n* = 50)	WOA group (*n* = 50)	*P*-value	95% CI
Remarkable bradycardia, *n* (%)	3 (6%)	11 (22%)	0.021*	0.057, 0.847
Hypotension, *n* (%)	1 (2%)	6 (12%)	0.112	0.017, 1.292
Delayed emergence, *n* (%)	3 (6%)	0 (0%)	0.242	0.876, 1.008
Dizziness, *n* (%)	6 (12%)	5 (10%)	0.749	0.349, 4.316
PONV, *n* (%)	2 (4%)	5 (10%)	0.436	0.069, 2.031

PONV, postoperative nausea and vomiting. *P < 0.05; **P < 0.01; ***P < 0.001.

## Discussion

This was a single-center, double-blind, randomized controlled trial. For the first time, the impact of OFA on the postoperative recovery quality of patients undergoing knee arthroscopy for military training-related injuries was investigated. The QoR15 scale, which evaluates recovery across five dimensions (pain, physical comfort, physical independence, psychological support, and emotional state), is simpler and more convenient compared to the QoR 40 scale. At 24 h postoperatively, the median difference in QoR15 scores between the two groups was 7, which exceeded the minimal clinically important difference of 6 and was thereby considered clinically significant. Although significant differences were also observed at 48 and 72 h, the median differences at these time points were <6.

Previous studies (15, 16) have shown that OFA can reduce the incidence of PONV, and Wang et al. (17) reported that OFA improved recovery quality by reducing the incidence of postoperative PONV. However, in this study, the incidence of PONV was similar between the groups. This may be explained by the homogeneity of gender (male participants), the type of surgery, and the use of certain medications (dexmedetomidine, dexamethasone, and ondansetron). In addition, the difference in recovery quality at 24 h was primarily reflected in the domains of physical comfort, emotional state, and pain. Similar to the findings of other OFA studies (18), the resting NPRS score in the OFA group was lower than that in the WOA group at 24 h after surgery. This suggests that postoperative pain may have contributed to the differences in recovery by influencing both physical comfort and emotional state. NPRS score gradually decreased on postoperative days 2 and 3, and the impact of pain on comfort and psychological state correspondingly weakened. This may explain why the differences in QoR15 score between the groups at 48 and 72 h after surgery were smaller than the minimal clinically important difference.

It is noteworthy that most previous OFA studies compared OFA with standard opioid-based anesthesia regimens, while this study compared OFA with weak opioid anesthesia. Alfentanil, characterized by its rapid onset and short duration of action, was selected in order to reduce the impact of opioid-related side effects on recovery quality. To further reduce residual neuromuscular blockade, rocuronium bromide was replaced with mivacurium chloride. Compared with standard opioid anesthesia, the medication regimen used in the WOA group was more consistent with the principles of ERAS. Therefore, the comparison between the OFA and WOA groups was also clinically significant.

Esketamine is a potent intravenous analgesic that plays an important role in opioid-free anesthesia and produces effective analgesia at low doses. Compared with ketamine, esketamine is associated with a shorter awakening time, but its potential relationship with delirium cannot be overlooked. The plasma concentration of ketamine during awakening from general anesthesia ranges from 600 to 1,100 ng/mL (19, 20), and its hallucinogenic effects are linearly related to steady-state plasma concentrations of 50–200 ng/mL (21). This finding indicates that even when the plasma concentration falls below the awakening threshold, significant psychiatric symptoms may still occur, especially in adults (22). However, no significant psychiatric symptoms were observed during the awakening period in this study, and there was no significant difference in 4AT scores between groups. This phenomenon may be attributed to the relatively small dose of esketamine (0.2 mg/kg), discontinuation of the medication 20 min before the end of surgery, and the concurrent infusion of dexmedetomidine (23), propofol, and sevoflurane (24). Norketamine, a metabolite of esketamine, has a half-life of 6–10 h and retains partial analgesic efficacy via NMDA receptor binding. This may explain the lower postoperative NPRS scores and reduced need for rescue analgesia in the OFA group. Rebound pain, defined as an increase in NPRS score from ≤3 to ≥7 within 24 h after nerve block (25), can cause considerable discomfort. The incidence of rebound pain was lower in the OFA group, which may be attributed to the preventive analgesia strategy (oral administration of loxoprofen sodium 2 h postoperatively) (26, 27) and the analgesic effect of esketamine (28). Notably, secretions increased significantly in the OFA group, which may have been related to esketamine. Therefore, airway suction is recommended to minimize the risk of airway obstruction.

Opioids exert a strong depressant effect on the circulatory system. This was reflected in the WOA group, which demonstrated lower intraoperative HR and MAP, along with a higher incidence of bradycardia and hypotension. The varying rates of bradycardia reported in earlier studies are likely related to differences in dexmedetomidine dosage, as bradycardia is strongly associated with higher doses of dexmedetomidine (29–31). In the present study, owing to long-term physical training, the subjects exhibited enhanced cardiac function and elevated vagal tone, often manifesting as a resting HR of 50–60 beats per minute. Consequently, only remarkable bradycardia was recorded, which was defined as HR ≤ 40 beats per minute. Interestingly, we found that the incidence of bradycardia in the OFA group was significantly lower than that in the WOA group, which may be related to the sympathetic excitability of esketamine and the dosage of dexmedetomidine. Similar to the findings of Beloeil and Garot (32), this study observed significantly longer awakening times in the OFA group. This delay may have resulted from the combined use of multiple sedative agents in the OFA group, leading to a superimposed effect.

This study has several limitations. First, being a single-center investigation, multicenter studies are warranted to validate these findings. Second, the subjects were exclusively middle-aged male soldiers, and therefore, further research is needed to establish the generalizability of these results to broader populations. Third, long-term outcomes were not assessed due to some constraints. Future studies should incorporate extended follow-up to evaluate the effect of OFA on long-term lower limb functional recovery.

## Conclusion

In summary, this study demonstrated that OFA improved the postoperative quality of recovery in military personnel undergoing meniscus surgery under general anesthesia at 24 h postoperatively. However, OFA was also associated with delayed emergence, which may be attributed to polypharmacy within the anesthetic regimen.

## Data Availability

The raw data supporting the conclusions of this article will be made available by the authors, without undue reservation.
