# Correction: Comparison of opioid-free versus weak-opioid general anesthesia on quality of postoperative recovery in soldiers undergoing arthroscopic meniscal surgery

**DOI:** 10.3389/fmed.2026.1774918

**Published:** 2026-01-29

**Authors:** Xikun Sun, Xin Ding, Xiaohang Han, Yue Wang, Xuli Cheng, Lin Li

**Affiliations:** 1 Department of Anesthesiology, General Hospital of Northern Theater Command, Shenyang, Liaoning, China; 2 Department of Anesthesiology, General Hospital of Northern Theater Command, Shenyang, Liaoning, China; 3 Graduate School, Dalian Medical University, Dalian, Liaoning, China

**Keywords:** opioid-free anesthesia, weak opioid anesthesia, QoR15 scores, soldier, meniscus surgery

In the published article, there was an error in Table 1 as published. The 95% confidence interval (CI) for postoperative first flatus was incorrectly reported as −2.416, 0.224. The corrected Table 1 and its caption [Demographics and procedure features] appear below.

**Table 1:** Demographics and procedure features.

	**OFA group (*n* = 50)**	**WOA group (*n* = 50)**	***P*-value**	**95% CI**
Male, *n* (%)	50 (100%)	50 (100%)		
Age (years)	28 (23.75, 32.25)	29 (24,33)	0.198	−4.546, 0.626
BMI (kg/m^2^)	24.11 ± 1.68	23.89 ± 1.81	0.855	−4.771, 0.909
ASA	23/27	25/25	0.689	1.005, 1.225
Surgery duration (min)	75 (65,85)	75 (65,87.5)	0.551	−10.564, 3.564
Anesthesia duration (min)	100 (85,111)	100 (88.75,115)	0.397	−12.590, 4.590
Wake up duration (min)	20 (17,23)	14 (12,15)	0.000^***^	5.311, 8.369
Postoperative first flatus (h)	11 (9,12.25)	12 (10,14)	0.03^*^	−2.416, −0.224
4AT at PACU discharge	0 (0,0)	0 (0,0)	0.31	−0.038, 0.118

In the published article, there was an error. The dosage of alfentanil was incorrectly listed as 0.2 μg/kg.

A correction has been made to Abstract (Method, Paragraph 2) and Methods (Study design, Paragraph 1).

The corrected sentence appears below:

“Anesthesia induction consisted of alfentanil 20 μg/kg in the WOA group and esketamine 0.2 mg/kg in the OFA group.”

“In addition, the OFA group received 2 mg/kg esketamine (Jiangsu Hengrui Pharmaceutical Co., Ltd.), while the WOA group received 20 μg/kg alfentanil (Yicahng Humanwell Pharmaceutical Co., Ltd.).”

The original version of this article has been updated.

